# An Interesting Case of X-linked Hypohidrotic Ectodermal Dysplasia

**DOI:** 10.7759/cureus.71014

**Published:** 2024-10-07

**Authors:** Alexander J Nardone, David S Kirwin, Willis H Lyford

**Affiliations:** 1 Department of Medical Education, Naval Medical Center San Diego, San Diego, USA; 2 Department of Dermatology, Naval Medical Center San Diego, San Diego, USA

**Keywords:** genetic skin disorders, genetic test, hypohidrotic ectodermal dysplasia, hypotrichosis, pilonidal cyst

## Abstract

We report a case of a patient with genetic sequencing-confirmed X-linked hypohidrotic ectodermal dysplasia without the typical characteristic hair growth abnormalities with the disorder. While this patient had already received guidance from a genetic counselor about his condition, many cases of ectodermal dysplasia go underdiagnosed or misdiagnosed due to mild or atypical presentations. With gene therapies emerging, the authors hope to highlight the importance of recognizing the disorder in patients who have not yet received a diagnosis to better manage their clinical course and guide future life decisions.

## Introduction

Hypohidrotic ectodermal dysplasia (HED) refers to a family of genetic disorders that affects the development of at least two tissues derived from the ectoderm during embryonic development [1]. Ectodermal derivatives include hair, teeth, nails, sweat glands, and the mouth. HED is a genetically heterogeneous disorder, with X-linked mutations being the most common form [2]. Patients with HED commonly share a characteristic triad of sparse hair growth (hypo/atrichosis), abnormal or missing teeth (hypo/anodontia), and decreased or absent sweating (hypo/anhidrosis) [1]. This case report describes a patient with genetically confirmed X-linked HED type 1 who presented with atypical features as a means to highlight the heterogeneity of this genetic disorder.

## Case presentation

An 18-year-old male presented to the dermatology clinic on referral from general surgery for laser hair reduction, as the patient had a history of two previously excised pilonidal cysts in the gluteal cleft. He had a secondary concern of premature balding, having previously used topical minoxidil inconsistently in the past. He had a tertiary concern of reduced sweating. He had recently completed whole genome sequencing, which showed a hemizygous variant in the EDA gene associated with X-linked HED type 1, and subsequently met with a genetic counselor to discuss his results.

**Figure 1:**
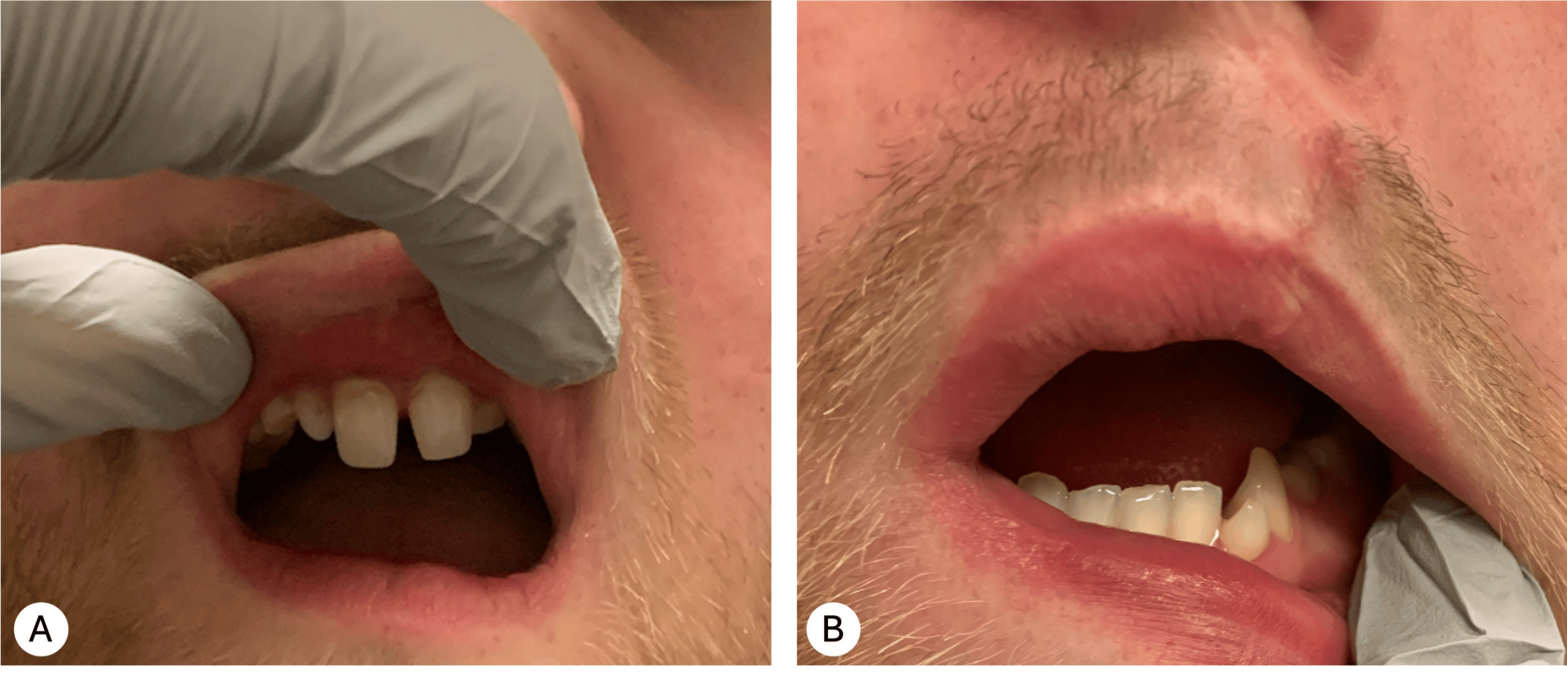
Photo of the patient’s dentition demonstrating peg-shaped upper central incisors (A) and conical lower left canine (B), a hallmark finding of hypohidrotic ectodermal dysplasia. Note that the patient has robust facial hair growth along with full eyebrows.

**Figure 2:**
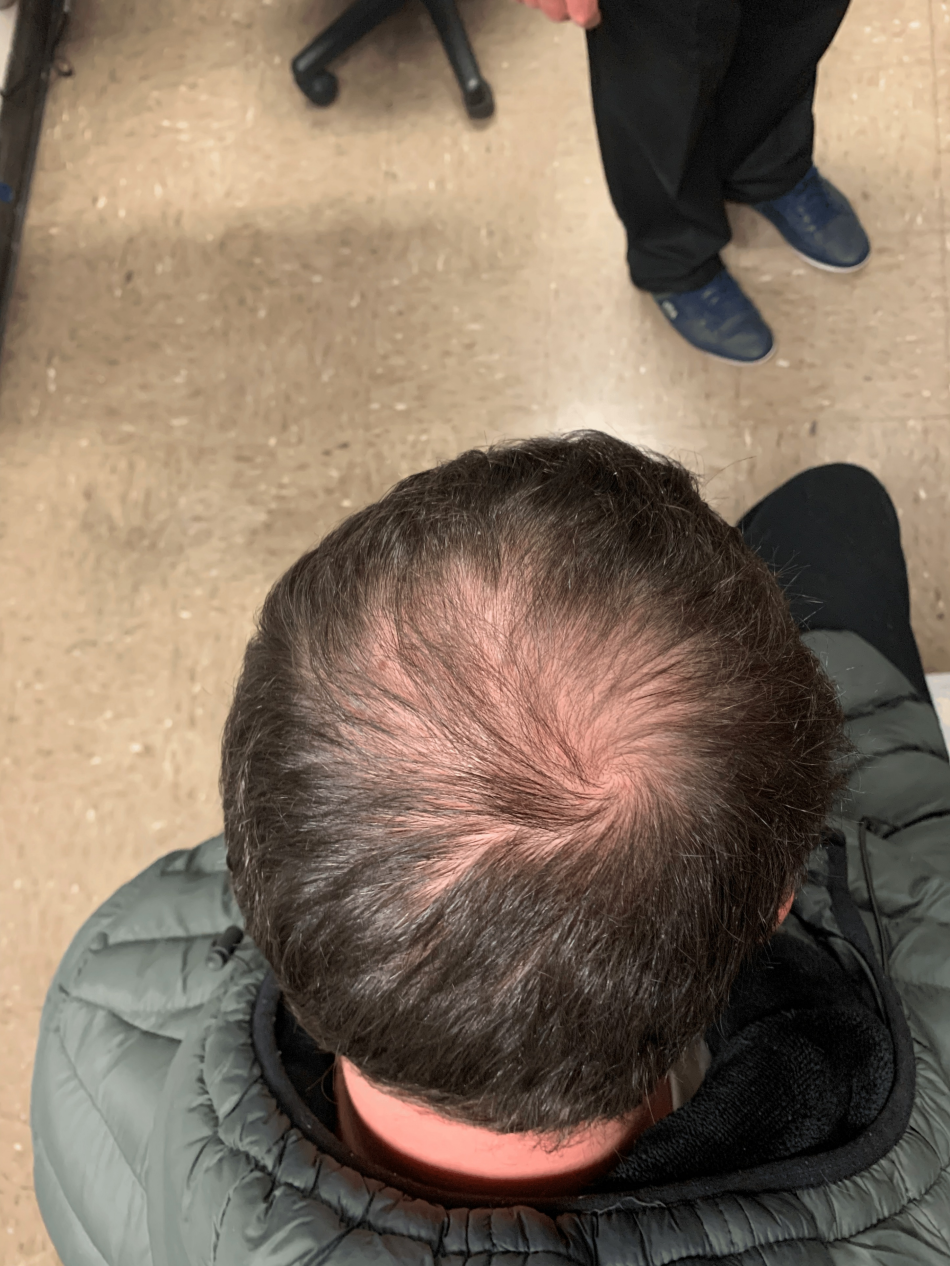
Photo of the patient’s scalp showing decreased hair density of the vertex scalp, consistent with androgenic alopecia, but otherwise healthy-appearing hair without the thin, brittle, sparsely distributed hairs seen in typical hypohidrotic ectodermal dysplasia patients.

**Figure 3:**
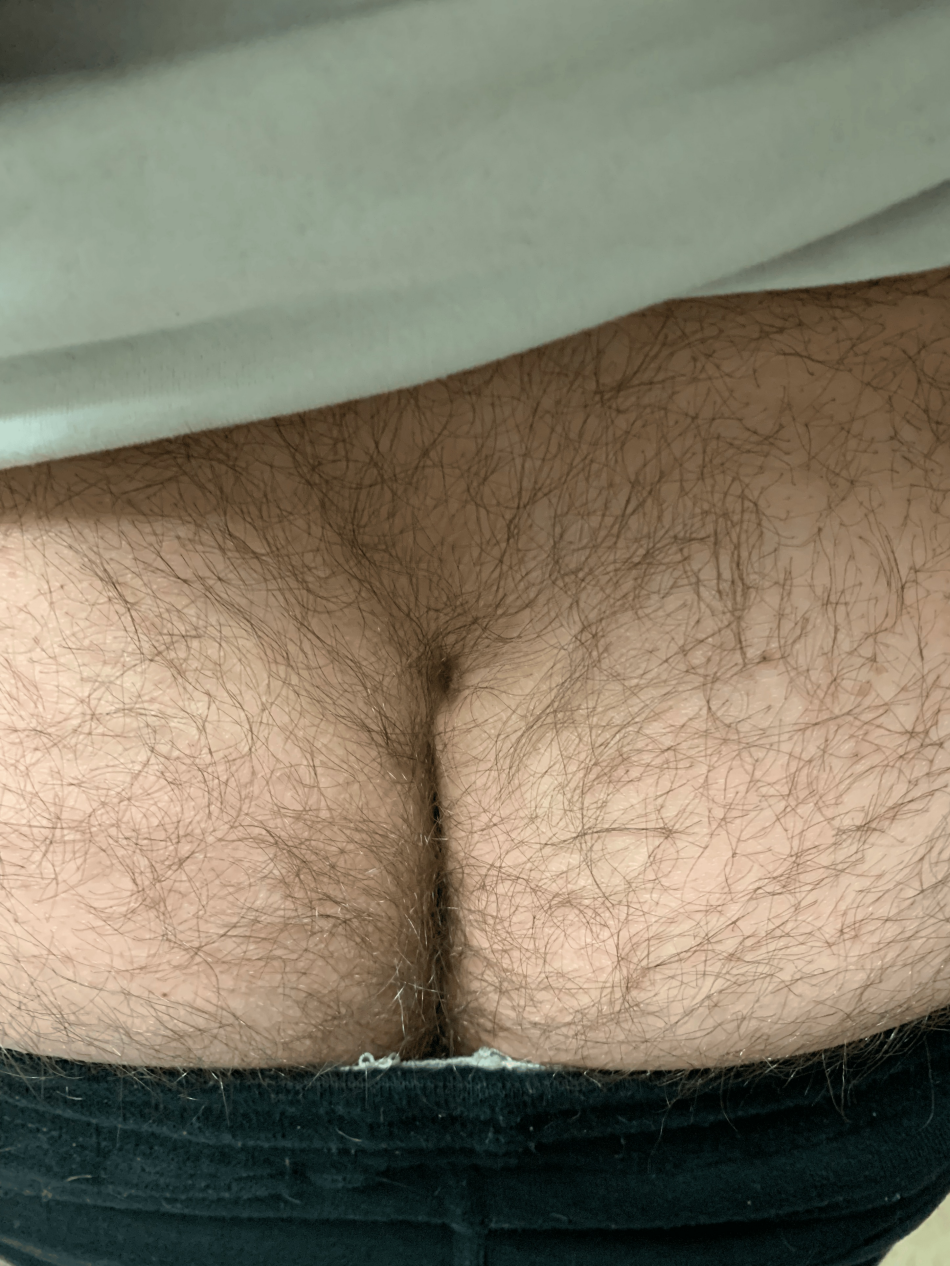
Photo of terminal hairs at the patient's gluteal cleft and buttock with a scar from previous pilonidal cyst excision visible at the superior pole.

**Figure 4:**
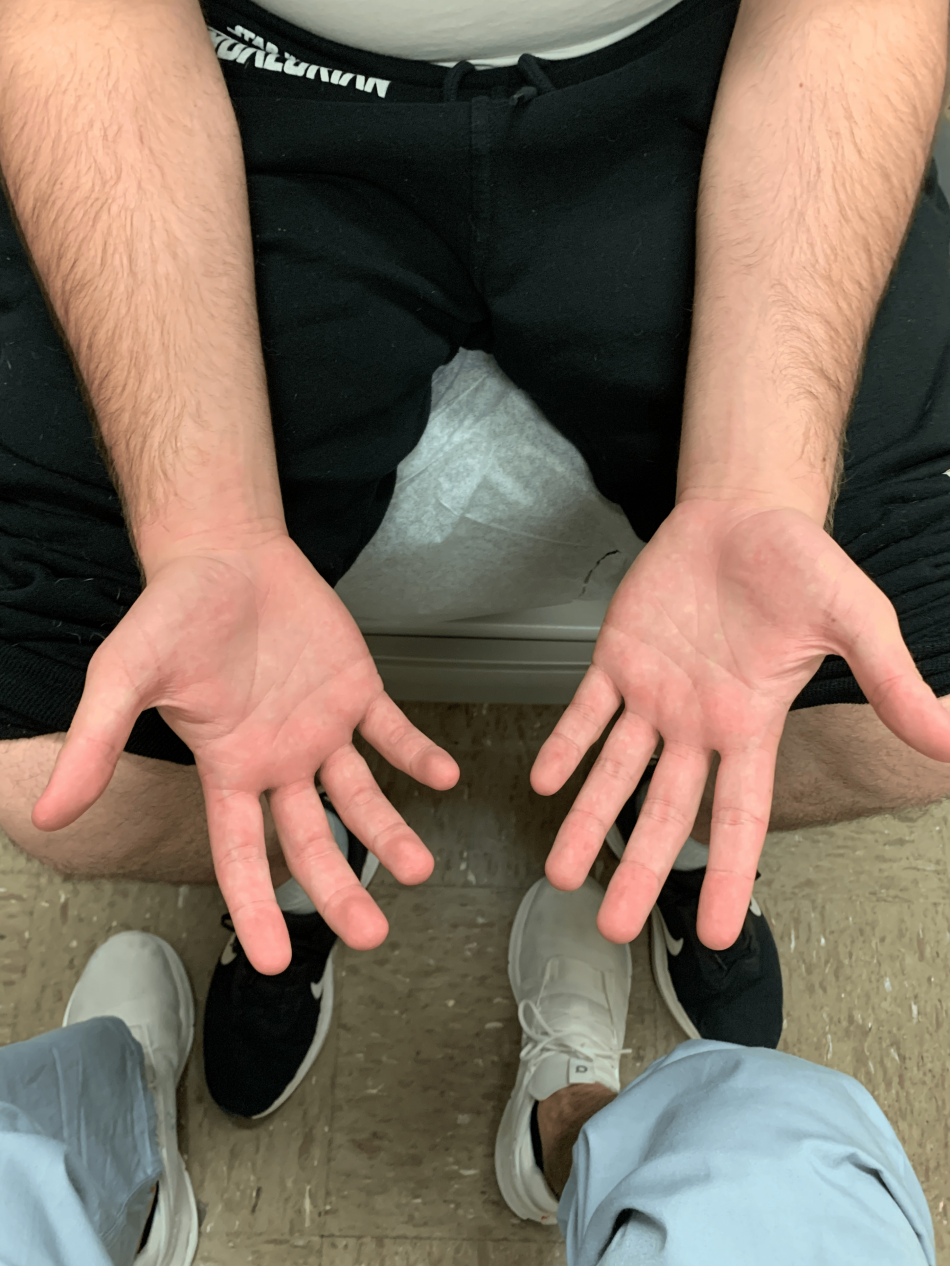
Photo of the patient’s arms and hands, which are notably lacking palmar hyperlinearity or signs of xerosis cutis.

Physical examination of the patient was notable for a light-skinned male with abnormally shaped and missing teeth (Figure 1), slightly coarse facial features, frontal bossing, malar hypoplasia, and dark wrinkled periorbital skin. There was a subtle scar on the upper lip from a previous cleft lip repair. There was decreased hair density of the frontal and vertex scalp (Figure 2) and dark terminal hairs along the upper gluteal cleft (Figure 3). Notably absent on physical examination were a lack of brittle hairs, a lack of xerosis cutis (although the patient and patient’s parent endorsed a history of eczema), and a lack of hyperlinearity of the palms and soles (Figure 4).

After a discussion of the risks and benefits of the procedure, the patient was scheduled for laser hair reduction of the gluteal cleft and was started on topical 5% minoxidil for androgenic alopecia.

## Discussion

Ectodermal dysplasias are a heterogeneous set of genetic disorders for which over 180 types and 49 molecular variants have been identified [3,4]. X-linked HED (XLHED) is the most common form of the disorder and is caused by mutations in the EDA gene, which results in the disruption of normal differentiation in ectodermal appendages [2]. Prevalence among the population is thought to be 22 people per 100,000 and roughly two per 100,000 when considering molecularly confirmed XLHED [5]. Typical HED is identified through the presence of the triad of hypotrichosis, hypodontia, and hypohidrosis. However, the phenotype of HED can be mild, resulting in a subset of patients going undiagnosed or misdiagnosed with a non-syndromic disorder of ectodermal derivatives and an underestimation of true prevalence within the population [4]. Males are primarily affected as a consequence of X-linked inheritance, but female carriers may demonstrate some features of the disorder due to random X-inactivation [6].

This patient presented with the typical hypodontia with peg-shaped teeth and a history of hypohidrosis. However, the characteristic slow-growing, thin, often lightly pigmented hair typical among patients with XLHED was curiously absent in this patient. Although he had overall reduced hair growth on the scalp, the distribution was instead consistent with androgenic alopecia. The patient also had full eyebrows and eyelashes, which are often absent in case reports [2]. The patient’s robust secondary sexual hair growth, including a full beard and anogenital hair, is less surprising as it can be a normal finding in patients with HED [7]. There is an overall dearth of literature analyzing hypotrichosis in both males and females with XLHED, but from the data available, hypotrichosis or atrichosis is reported in 88.1% of males [8]. This suggests that while considered a cardinal feature of the disorder, a lack of hypotrichosis should not interfere with the pursuit of genetic testing in patients with suspected ectodermal dysplasia. This is especially important today, as novel prenatal treatments with recombinant fusion proteins consisting of the receptor-binding domain of EDA and the Fc protein of IgG1 have been shown to mitigate or prevent symptomology through at least 22 months of age [9]. Many parents with a child who has an inherited genetic disorder report mixed feelings about having another child, with a majority choosing not to conceive further [10]. Indeed, previous investigations into reproductive decision-making of women with known carrier status of XLHED have shown that these women have moral conflicts with pregnancy, which causes some to actively avoid pregnancy altogether [11]. Carriers of and patients with XLHED can be reassured by their clinicians that more reproductive options are on the horizon for them.

## Conclusions

XLHED can have a variable presentation in those with the mutation, which can make diagnosis in patients without confirmed genetic testing difficult. Clinicians should be aware of the heterogeneic presentation of this disorder to provide better care, counseling, and management of these patients. With prenatal gene therapy now possible in humans, timely diagnosis is critical to assist with mitigating symptoms in a patient’s future offspring.

## References

[1] Singh GP, Saxena V (2015). Hypohidrotic ectodermal dysplasia. Med J Armed Forces India.

[2] Yu K, Shen Y, Jiang CL, Huang W, Wang F, Wu YQ (2021). Two novel ectodysplasin A gene mutations and prenatal diagnosis of X-linked hypohidrotic ectodermal dysplasia. Mol Genet Genomic Med.

[3] Itin PH (2014). Etiology and pathogenesis of ectodermal dysplasias. Am J Med Genet A.

[4] Peschel N, Wright JT, Koster MI (2022). Molecular pathway-based classification of ectodermal dysplasias: first five-yearly update. Genes (Basel).

[5] Nguyen-Nielsen M, Skovbo S, Svaneby D, Pedersen L, Fryzek J (2013). The prevalence of X-linked hypohidrotic ectodermal dysplasia (XLHED) in Denmark, 1995-2010. Eur J Med Genet.

[6] Alikhan A, Hocker TLH (2016). Review of Dermatology, 2nd Edition. Review of Dermatology, 2nd Edition.

[7] Wright JT, Grange DK, Fete M (2024). Hypohidrotic ectodermal dysplasia. GeneReviews®.

[8] Anbouba GM, Carmany EP, Natoli JL (2020). The characterization of hypodontia, hypohidrosis, and hypotrichosis associated with X-linked hypohidrotic ectodermal dysplasia: a systematic review. Am J Med Genet A.

[9] Schneider H, Faschingbauer F, Schuepbach-Mallepell S (2018). Prenatal correction of X-linked hypohidrotic ectodermal dysplasia. N Engl J Med.

[10] Kelly SE (2009). Choosing not to choose: reproductive responses of parents of children with genetic conditions or impairments. Sociol Health Illn.

[11] Leo B, Schneider H, Hammersen J (2022). Reproductive decision-making by women with X-linked hypohidrotic ectodermal dysplasia. J Eur Acad Dermatol Venereol.

